# Adjunct N-Acetylcysteine Treatment in Hospitalized Patients With HIV-Associated Tuberculosis Dampens the Oxidative Stress in Peripheral Blood: Results From the RIPENACTB Study Trial

**DOI:** 10.3389/fimmu.2020.602589

**Published:** 2021-02-04

**Authors:** Izabella P. Safe, Eduardo P. Amaral, Mariana Araújo-Pereira, Marcus V. G. Lacerda, Vitoria S. Printes, Alexandra B. Souza, Francisco Beraldi-Magalhães, Wuelton M. Monteiro, Vanderson S. Sampaio, Beatriz Barreto-Duarte, Alice M. S. Andrade, Renata Spener-Gomes, Allyson Guimarães Costa, Marcelo Cordeiro-Santos, Bruno B. Andrade

**Affiliations:** ^1^ Fundação de Medicina Tropical Dr. Heitor Vieira Dourado, Manaus, Brazil; ^2^ Programa de Pós-Graduação em Medicina Tropical, Universidade do Estado do Amazonas, Manaus, Brazil; ^3^ Immunobiology Section, National Institutes of Allergy and Infectious Diseases, National Institutes of Health, Bethesda, MD, United States; ^4^ Laboratório de Inflamação e Biomarcadores, Instituto Gonçalo Moniz, Fundação Oswaldo Cruz (FIOCRUZ), Salvador, Brazil; ^5^ Multinational Organization Network Sponsoring Translational and Epidemiological Research (MONSTER) Initiative, Salvador, Brazil; ^6^ Instituto Leônidas & Maria Deane, Fundação Oswaldo Cruz, Manaus, Brazil; ^7^ Curso de Medicina, Universidade Salvador (UNIFACS), Laureate Universities, Salvador, Brazil; ^8^ Curso de Medicina, Universidade Federal do Amazonas, Manaus, Brazil; ^9^ Curso de Medicina, Universidade Nilton Lins, Manaus, Brazil; ^10^ Curso de Medicina, Faculdade de Tecnologia e Ciências (UniFTC), Salvador, Brazil; ^11^ Curso de Medicina, Escola Bahiana de Medicina e Saúde Pública (EBMSP), Salvador, Brazil

**Keywords:** tuberculosis, oxidative stress, N-acetylcysteine treatment, glutathione, RIPENACTB

## Abstract

Tuberculosis (TB) still causes significant morbidity and mortality worldwide, especially in persons living with human immunodeficiency virus (HIV). This disease is hallmarked by persistent oxidative stress and systemic inflammation. N-acetylcysteine (NAC), a glutathione (GSH) precursor, has been shown in experimental models to limit *Mycobacterium tuberculosis* infection and disease both by suppression of the host oxidative response and through direct antimicrobial activity. In a recent phase II randomized clinical trial (RIPENACTB study), use of NAC as adjunct therapy during the first two months of anti-TB treatment was safe. Whether adjunct NAC therapy of patients with TB-HIV coinfection in the context of anti-TB treatment could directly affect pro-oxidation and systemic inflammation has not been yet formally demonstrated. To test this hypothesis, we leveraged existing data and biospecimens from the RIPENACTB trial to measure a number of surrogate markers of oxidative stress and of immune activation in peripheral blood of the participants at pre-treatment and at the day 60 of anti-TB treatment. Upon initiation of therapy, we found that the group of patients undertaking NAC exhibited significant increase in GSH levels and in total antioxidant status while displaying substantial reduction in lipid peroxidation compared to the control group. Only small changes in plasma concentrations of cytokines were noted. Pharmacological improvement of the host antioxidant status appears to be a reasonable strategy to reduce TB-associated immunopathology.

## Introduction

Tuberculosis (TB), a communicable disease caused by *Mycobacterium tuberculosis* (Mtb), remains a major public health challenge (1). It is estimated 10 million people were infected with Mtb and developed active TB worldwide in 2019, accounting for 1.2 million TB-associated deaths among HIV-negative people and additional 251,000 deaths among HIV/TB co-infected individuals (1). Persons living with HIV (PLWHIV) are highly susceptible to infection with opportunistic pathogens such as Mtb, and usually patients develop severe forms of TB due to their HIV-associated immune suppression (2). Although anti-TB drug regimens have shown considerable efficacy against drug-sensitive Mtb strains, increases in drug-resistant TB has been reported and causes elevated mortality, especially among PLWHIV (3–5). These observations indicate that a new adjunctive therapy strategy is crucial to enhance the effectiveness of the current TB treatment protocol mainly for those HIV-affected subjects.

Intracellular glutathione (GSH) level has been pointed as an important biomarker in HIV infection (6). A number of studies have shown that macrophages obtained from PLWHIV display drastic depletion of intracellular GSH levels, favoring the growth of opportunistic pathogens such as Mtb (7, 8). Another consequence of GSH depletion is the inability of host cells in controlling the generation of reactive oxygen species (ROS). The accumulation of ROS in the context of TB is frequently reach levels that are toxic to host immune cells (9). Interestingly, the treatment of macrophages isolated from PLWHIV with n-acetylcysteine (NAC), a precursor of GSH known to restore intracellular GSH levels, has been shown to improve macrophage microbicide capacity to control Mtb infection (10). Furthermore, NAC has been shown to diminish intracellular ROS levels and thus improving human monocyte-derived macrophages to control Mtb infection (11). In addition, NAC treatment when given to mice infected with Mtb enhances host capacity in controlling Mtb growth in the lungs (11), which has also been observed in other animal models (12). Recently, several clinical studies utilizing NAC as adjunct therapy to TB have shown promising data revealing a potential use for this drug as host-directed therapy (HDT) in TB (13–16).

Besides NAC effects on Mtb growth, several studies have suggested this drug as an important modulator of host cell biology and immune response (17–20). NAC has been used to prevent ferroptosis, a regulated form of lipid ROS-mediated necrotic cell death, in several models (18, 21). The efficacy in modulating cell fate is attributed to capacity of NAC to restore intracellular levels of GSH, preventing the accumulation of toxic lipid peroxides and consequently cells to undergo necrotic cell death. Our group and others have shown that NAC prevents Mtb-induced macrophage death (11, 22–24). In addition, we have recently shown that Mtb-induced necrosis is associated with reduced levels of intracellular GSH and elevated ROS generation, which were diminished when Mtb-infected macrophage cultures were treated with either iron chelator or ferrostatin-1, both drugs known to prevent ferroptosis (25). Moreover, NAC has been shown to modulate host immune response ensuring host capacity to control infection, and thus preventing exacerbation of inflammatory responses against pathogens (26, 27). These results from experimental models are encouraging; however, whether treatment with NAC in TB patients directly leads to modulation of systemic oxidative stress and immune activation is still unknown.

Recently, we have reported a clinical trial conducted with hospitalized HIV/TB patients in Brazil in which we evaluated the safety of NAC and its efficacy in accelerating mycobacterial clearance in sputum cultures (16). In the present study, we leveraged existing data and biospecimens from the RIPENACTB trial to assess the impact of NAC adjunctive treatment on host immune response and redox homeostasis in this population of hospitalized patients with HIV-associated TB. We measured several surrogate markers of oxidative stress and of immune activation in peripheral blood of the participants at pre-treatment and at the day 60 of anti-TB treatment. Our findings demonstrated that patients receiving NAC displayed significant increase in GSH levels and total antioxidant status along with a strong reduction in lipid peroxidation and DNA oxidation in comparison with those who did not undertake NAC. Thus, we suggest that NAC may be considered as a potential drug for adjunctive TB antibiotic therapy aid preventing TB-associated immunopathology related to oxidative stress.

## Methods

### Ethics Statement

The study was approved by the Ethics Review Committee of Fundação de Medicina Tropical Dr Heitor Vieira Dourado (FMT-HVD) (protocol study number: 60219916.5.0000.0005). Written informed consent was obtained from all participants (or relatives in case of unconscious patients), after detailed information about the study protocol was given. The parent randomized clinical trial is registered at ClinicalTrials.gov (Identifier: NCT03281226).

### Study Design, Participants, and Treatments

The present study is a prospective investigation which was nested with the recently published RIPENACTB Study trial (16). Detailed description of study design, inclusion and exclusion criteria, randomization and study treatments is presented in our previous publication (16). The RIPENACTB Study trial was an open-label, single center, randomized, phase II trial to test whether NAC-containing treatment regimen was as safe as the standard regimen for TB treatment in hospitalized patients with HIV. The study also explored the potential treatment efficacy by means of culture conversion of sputum samples. The study was conducted at FMT-HVD, which is a tertiary care reference public institution for TB/HIV coinfection in Manaus, Brazilian Amazon, between December 2016 and April 2018.

Persons living with HIV from both sexes, age ≥ 18 years, with diagnosis of pulmonary TB confirmed through positive Xpert-Mtb/RIF, and who were hospitalized (at clinician’s discretion) for more than 24 hours, were eligible to be included in the study. Patients without HIV, with extrapulmonary TB only, who were unable to collect respiratory sample, pregnant and lactating women, exposed to quinolones in the last 7 days, and in use of anti-TB drugs for more than 72 hours or in use of anti-TB drugs as second line drugs were not included in the study. Enrolled patients were subsequently excluded if their baseline culture (Mycobacteria Growth Indicator Tube, MGIT) failed to grow Mtb or grew a strain of Mtb that was resistant to any anti-TB drug (16).

Study participants were randomized into control group (RIPE) or NAC group (RIPENAC) in a 1:1 ratio using a computer-generated randomization table. The groups received standard anti-TB treatment with RIPE (rifamycin 150 mg, isoniazid 75 mg, pyrazinamide 400 mg, ethambutol 275 mg), fixed dose tablets combined according to weight, for 8 weeks. RIPE was supplied by Farmanguinhos, Rio de Janeiro, Brazil. In addition, patients who enrolled the NAC group received two effervescent tablets containing N-acetylcysteine (Fluimucil^®^) 600 mg bid, for 8 weeks. Tablets were dissolved in water before oral ingestion or administration though the nasoenteral tube (16).

The study participants and the assisting infectious disease physicians were aware of the treatments, except laboratory team which performed the clinical laboratory and the immunoassays presented in the present study, to whom the study was blinded (16). During hospitalization, all the medication was administered in a supervised way by the nursing team. After discharge, patients were asked to take anti-TB drugs following the guidelines from the Brazilian TB program (28), and NAC only until 8 weeks was completed. During every visit to the clinics, patients were requested to bring medication packages for tablet counting, as a proxy of adherence (16).

All study participants underwent a baseline evaluation, which included physical examination, sputum (spontaneous or induced whenever sputum production was considered insufficient) or tracheal aspirate in unconscious patients, CD4+ T-lymphocyte count, HIV viral load, serum levels of aspartate aminotransferase (AST), alanine aminotransferase (ALT), bilirubins, and screening of concomitant drug exposures and chest radiograph, as previously described (16). Safety assessments were performed at baseline (before treatment) and weeks 1, 2, 4, 6, and 8. Additional exams were solicited whenever needed. EDTA plasma and serum samples were collected at study baseline (before treatment) and at day 60 of therapy and cryopreserved in −80°C until used for colorimetric and immune-based biomarker assays.

Respiratory samples were submitted to smear Ziehl–Nielsen staining technique, Xpert-Mtb/RIF for Mtb, and sown in liquid culture BACTEC MGIT™ 960 and solid culture Löwenstein-Jensen as previously described (16).

### Laboratory Measurements

Serum samples were used to measure total oxidant status, total antioxidant status and glutathione levels. Total oxidant was assessed using an enzymatic assay kit from Rel Assay Diagnostics (Gaziantep, Turkey) following the manufacturer’s protocol. The results are expressed in terms of micro molar hydrogen peroxide equivalent per liter (μmol H_2_O_2_ Equiv./L). Total antioxidant status was measured using the Antioxidant Assay kit from Cayman Chemical (Ann Harbor, MI). In this assay, the capacity of the antioxidants to prevent ABTS (2,2′-azino-di-[3-ethylbenzthiazoline sulphonate]) oxidation is compared with that of Trolox, a water-soluble tocopherol analogue, and is quantified as molar Trolox equivalents. The other analyses were performed using cryopreserved plasma samples. Lipid peroxidation in plasma was quantified using an assay kit from Cayman Chemical, which measures the formation of malondialdehyde (MDA). Concentration of glutathione (GSH), superoxide dismutase activity (SOD), prostaglandin E2 (PGE2) and leukotriene B4 (LTB4) were measured using kits from Cayman Chemical following the manufacturer’s protocol. The inclusion of both PGE2 and LTB4 in the list of analytes was based on previous work from our group demonstrating the participation of these lipid mediations in TB-associated pathology and disease severity (29). DNA oxidation was measured using an immunoassay from Cayman Chemical that detects 8-hydroxy-2′-deoxyguanosine. Levels of ferritin heavy chain (ferritin-H) were measured using an ELISA kit (Abnova, Taipei City, Taiwan). Concentrations of catalase were assessed using a colorimetric assay from Thermo Fisher (Waltham, MA).

A large panel of cytokines, chemokines and growth factors was measured using a pre-defined, commercially available, Luminex kit from Millipore (Burlington, MA) which included the cytokines interleukin (IL)-1α, IL-1β, IL-1RA, IL-2, IL-3, IL-4, IL-6, IL-7, IL-8, IL-10, IL-12p40, IL-12p70, IL-15, IL-17A, interferon (IFN)-α2, IFN-γ, tumor necrosis factor (TNF)-α, TNF-β, granulocyte colony-stimulating factor (GCSF), granulocyte-macrophage colony-stimulating factor (GMCSF), epidermal growth factor (EGF), vascular endothelial growth factor (VEGF), monocyte chemoattractant protein-1 (MCP1/CCL2), IFN-γ–induced protein/chemokine (C-X-C motif) ligand 10 (IP10/CXCL-10), macrophage inflammatory protein-1 alpha (MIP1α/CCL3), MIP-1 beta (MIP1β/CCL4), and eotaxin (CCL11). These cytokines, lipid mediators and growth factors were evaluated to examine the overall profile of inflammation and immune activation. The selection of analytes was based on potential role in TB pathogenesis demonstrated by previous studies from our group (29–35) and others (36, 37). The selection of the analytes was also based on availability of the parameters in pre-mixed Luminex kits that could assume measurements using the same range of sample dilution.

The quality control of each analyte included the following criteria: (i) the standard concentrations needed to be within 80% to 110% of their expected values; (ii) the accepted coefficient of variation between replicated samples was ≤10%. Values that were under the limit of detection were inputted as zero or 0.01 (to be log10-transformed in heatmaps). No values above the upper limit of detection were obtained.

### Statistical Analysis

Descriptive statistics were performed to characterize the study population. Continuous variables were tested for Gaussian distribution using the D’Agostino-Pearson test. The median values with interquartile ranges (IQR) were used as measures of central tendency and dispersion, respectively, for parameters which values exhibited a non-Gaussian distribution. For variables which values displayed a Gaussian distribution in the study population (log10-transformed Luminex data) mean and 95% confidence intervals were used as measures of central tendency and dispersion. All the statistical analyses were pre-specified. The Mann-Whitney *U* test was used to compare values at day 60 between RIPE and RIPENAC groups whereas the Wilcoxon matched pairs test was employed to compare values measured at day 0 with those from day 60 within each study group. Differences with p-values below 0.05 after Holm-Bonferroni’s adjustment for multiple comparisons were considered statistically significant.

To evaluate the overall profile of cytokines, an unsupervised hierarchical was performed using the Ward’s method. In this analysis, the dendrograms represent the Euclidean distance (inferring degree of similarity). To plot heatmaps, the concentrations of each marker were log10 transformed and normalized using Z-score method. The Z-score is a strategy of normalizing data using the following formula: (value of the analyte in a given group subtracted by the mean of the analyte concentration in all the groups) divided by the standard deviation of the analyte value in all the groups. To link the clusters of inflammatory markers with immunological pathways, a pathway enrichment was performed using the gene related to the markers present in each cluster. The list of gene markers was applied to the *Enrichr* web tool (https://amp.pharm.mssm.edu/Enrichr/), that returns any enrichment of common annotated biological features. To define the pathways associated to clusters was used a cut-off of adjusted p-value less than 0.05 on the *WikiPathways* database. *WikiPathways* is a database of biological pathways maintained by the scientific community with peer review and editorial curation (38). Each pathway of this database has an identification code that starts with WP followed by its registration number (i.e., WP1234).

## Results

### Effect of NAC Therapy in Modulating Cytokines, Chemokines, Growth Factors, and Lipid Mediators in Hospitalized HIV/TB Patients

The randomized clinical trial was conducted between December 2016 and April 2018 with a total of 39 patients eligible for this study where 21 individuals were included in the control group (referred as “RIPE”) and 18 patients were allocated in the NAC-treated group (referred as “RIPENAC”). The detailed description of the protocol used as well as demographic and clinical characteristics of participants have been described in a separate study previously published (16). In the parental study, we have shown the use of NAC-adjunctive therapy in hospitalized HIV/TB patients was safe and had a potential impact on faster negative conversion of Mtb cultures, suggesting a potential effect of NAC in controlling Mtb growth in these patients.

**Figure 1:**
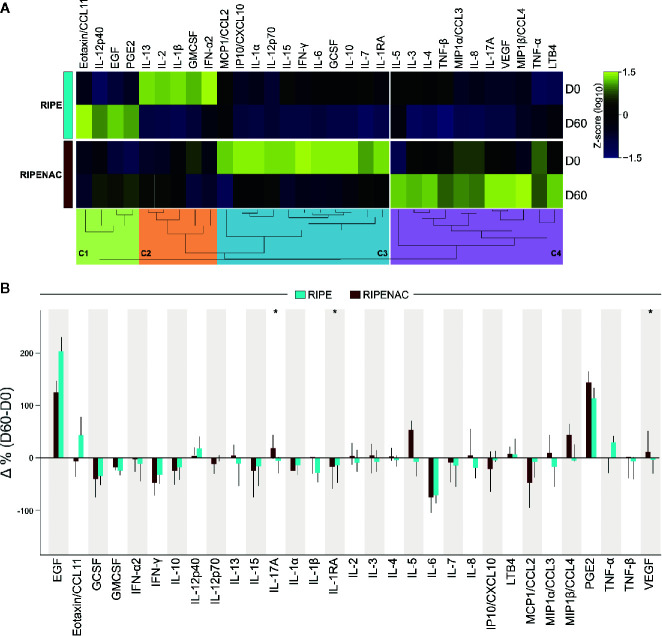
Dynamics of circulating levels of cytokines, chemokines, growth factors, and lipid mediators. **(A)** Plasma concentrations of the indicated mediators of inflammation were measured at the study baseline and at day 60 of antitubercular treatment ± NAC adjunct therapy using Luminex kit and immunoassays for PGE2 and LTB4. Median Log10 values of each study group and timepoint were z-score normalized and a heatmap with an unsupervised hierarchical cluster analysis was performed to illustrate the overall profiles of the biomarkers. In this analysis, dendrograms represent the Euclidean distance. Four major clusters of markers were identified (C1-C4) and enriched to, respectively, lung fibrosis (C1), cytokines and inflammatory response (C2 and C3) and selective expression of chemokine receptors during T-cell polarization (C4). **(B)** Delta percent variation (D60-D0) was calculated (median and 95% CI) for each marker and magnitude of changes were compared between the study groups (RIPE vs. RIPENAC) using the Mann-Whitney *U* test, where “*” indicates differences with p < 0.05.

To further assess the effect of NAC treatment in modulating host immune response, we measured the concentration of several cytokines, chemokines, growth factors and lipid mediators in plasma isolated from patients receiving or not NAC at the study baseline and day 60 of treatment (see Methods for details in the choice of the parameters). Plasma levels of 31 inflammatory mediators including cytokines, chemokines, growth factors and lipid mediators were compared between RIPE and control RIPENAC groups (**Figure 1**). The concentration values of each inflammatory mediators at baseline are described in **Table S1**. Prior to anti-TB treatment commencement, concentrations of most of the biomarkers were undistinguishable between the study groups. However, patients from the RIPENAC group did present with higher plasma levels of IL-1α (p = 0.034) and of IP10/CXCL10 (p = 0.025) than those from the RIPE group (**Table S1**).

To visualize overall trends in variation of the plasma concentrations of the inflammatory markers between the groups and timepoints, we plotted a heatmap. To do that, we normalized these data using Z-score method of each different group and timepoint and plotted an unsupervised hierarchical cluster analysis (see methods for details) which revealed four major clusters of inflammatory markers (C1-C4), as shown in **Figure 1A**. These clusters thus represented groups of biomarkers that exhibited a similar trend of variation in plasma concentrations between the clinical groups and timepoints. We observed that parameters included in clusters associated with lung fibrosis (WikiPathways [WP] 3624, p = 0.027) (cluster #1, C1) and cytokines and inflammatory response (WP530, p < 0.001) (cluster #2, C2) displayed heightened concentrations at the timepoints 0 and 60, respectively, on the RIPE group. The markers grouped in the cluster #3 (C3) correlated with a pro-inflammatory response (WP530, p < 0.001) and selective expression of chemokine receptors during T-cell polarization (WP4494, p < 0.001) (cluster #4, C4) (**Figure S1**); such subgroups of molecules exhibited higher relative concentrations in the group of patients receiving NAC therapy. The enrichment pathway analysis for each cluster of biomarkers is summarized in **Figure S1**. The variation in concentrations of each mediator of inflammation in plasma measured between baseline and day 60 of anti-TB therapy was next assessed. Within the RIPE group, we detected significant reduction in circulating concentrations of IFN-γ (p = 0.033) and of IL-6 (p = 0.007) at day 60 of treatment compared with those measured at pre-treatment timepoint (**Table S2**). In the RIPENAC group, levels of IL-17A (p = 0.05) increased whereas those of IL-1RA (p = 0.02) and IP10/CXCL10 (p = 0.048) reduced at day 60 compared with those assessed at day 0 (**Table S2**). Notwithstanding when we analyzed the delta variation between day 60 versus day 0 of each biomarker and compared both groups of patients, we found that the change in concentrations of three pro-inflammatory mediators were distinct between the clinical groups. Interestingly, IL1RA, which has been shown to be detrimental for host defense in TB (39), displayed negative variation in both study groups, with more robust reduction detected in those undertaking RIPENAC (RIPE: −13.8%, RIPENAC: −17.2%, p = 0.027). IL-17A (RIPE: −5%, RIPENAC: 19%, p = 0.012) and VEGF (RIPE: −2.94%, RIPENAC: 12.03%, p = 0.043) also presented significative differences in delta variation between the groups, but with a different profile characterized by increases detected in the RIPENAC group and reductions observed in the RIPE group. (**Figure 1B** and **Table S2**). In general, it seemed that the exacerbation of inflammatory immune response was better controlled in RIPENAC group compared to RIPE group, although the variation of these mediators analyzed in this study was not strongly remarkable.

### The Use of NAC as Adjunctive Therapy Ameliorates Redox Homeostasis Status and Cell DNA Damage in Hospitalized HIV/TB Patients

NAC is known as a precursor of the reduced form of GSH which is critical for the optimal activity of glutathione peroxidase-4 in controlling the generation of toxic forms of lipid peroxides. Thus, we wanted to investigate whether NAC-adjunct therapy may restore redox homeostasis in hospitalized HIV/TB patients. As parameters of redox homeostasis status, we measured GSH, total antioxidant status (determined by the concentration of trolox), total oxidant status (measured by the concentration of hydrogen peroxides), lipid peroxidation (evaluated by malondialdehyde [MDA] levels) and cell DNA damage (determined by levels of 8-Hydroxy-2′-deoxyguanosine [8-OHdG]) in plasma obtained from patients receiving or not NAC at two time-points (at baseline and day 60). We also evaluated the levels of other antioxidant molecules such as catalase, total superoxide dismutase activity, and ferritin heavy chain. Of note, the frequency of patients that received HAART in both groups was close to 50% and we were not able to observe significant differences comparing groups (RIPE: 42.85% vs. RIPENAC: 44.4%, p = 0.549) (16). Thus, HAART did not seem to affect the evaluation of antioxidant status and immune restoration in this study.

**Figure 2:**
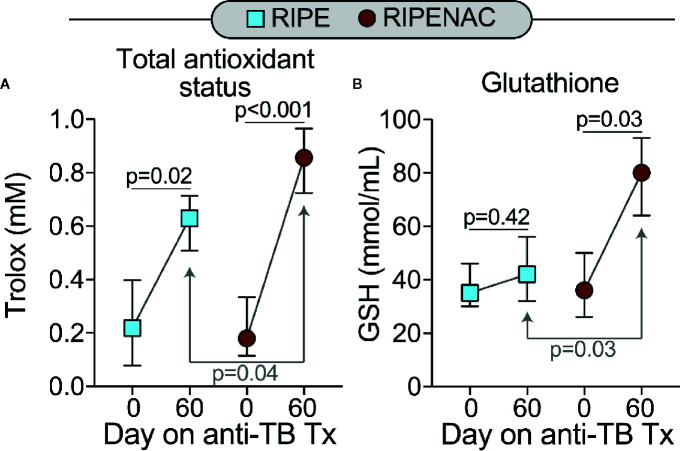
Adjunct NAC therapy is associated with higher levels of glutathione and values of total antioxidant status in plasma. Serum concentrations of **(A)** values of total antioxidant status and **(B)** glutathione (GSH) were measured using enzymatic assays described in Methods. Data represent median values and interquartile ranges. The Mann-Whitney *U* test was used to compare values at day 60 between RIPE and RIPENAC groups whereas the Wilcoxon matched pairs test was employed to compare values measured at day 0 with those from day 60 within each study group.

First, we investigated the impact of NAC treatment in modulating the total antioxidant status. We observed that TB therapy alone was capable to increase total antioxidant status as seen by comparing trolox levels at day 60 with values detected at baseline in both groups, with p = 0.02 in RIPE group and p < 0.001 in RIPENAC. However, stronger increase (p = 0.04) in total antioxidant status was found in patients receiving NAC compared to control group (**Figure 2A**). Interestingly, GSH levels were elevated only in RIPENAC patients at 60 days compared to levels found at baseline (p = 0.03). Furthermore, GSH levels were higher in RIPENAC patients compared to RIPE group at day 60 (p = 0.03) (**Figure 2B**). Despite the positive benefit of NAC treatment in raising levels of both total antioxidant status and GSH, it seems that this therapy was more efficient in enhancing GSH levels (~2-fold increase, RIPENAC vs RIPE at 60 days) than total antioxidant status (~1.3-fold increase, RIPENAC vs RIPE at 60 days) (**Figure 2**).

**Figure 3:**
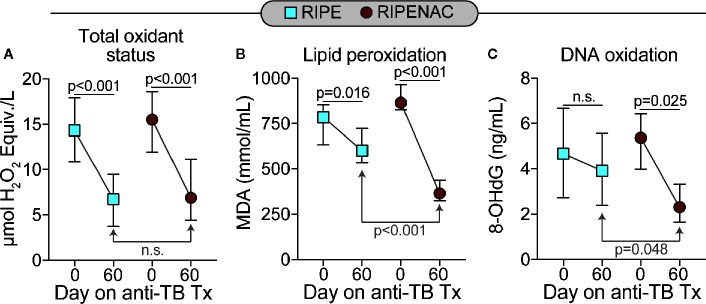
Decreased systemic oxidation in patients undertaking NAC adjunct therapy. Values of **(A)** total oxidant status, **(B)** lipid peroxidation (Malondialdehyde [MDA]) and of **(C)** 8-Hydroxy-2′-deoxyguanosine (8-OHdG, a surrogate of DNA oxidation) were measured using enzymatic assays described in Methods. Data represent median values and interquartile ranges. The Mann-Whitney *U* test was used to compare values at day 60 between RIPE and RIPENAC groups whereas the Wilcoxon matched pairs test was employed to compare values measured at day 0 with those from day 60 within each study group. ns, not significant.

**Figure 4:**
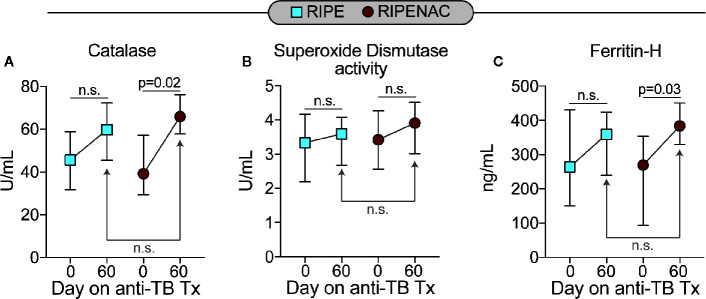
Low impact of antitubercular treatment on plasma concentrations of other antioxidants. Plasma values of **(A)** catalase, **(B)** total superoxide dismutase activity and of **(C)** ferritin heavy chain (ferritin-H) were measured using enzymatic assays described in Methods. Data represent median values and interquartile ranges. The Mann-Whitney *U* test was used to compare values at day 60 between RIPE and RIPENAC groups whereas the Wilcoxon matched pairs test was employed to compare values measured at day 0 with those from day 60 within each study group. n.s., nonsignificant (p ≥ 0.05).

Next, we measured the levels of total oxidant status as well as lipid peroxidation and DNA oxidation as a readout of cell stress and damage. TB drug therapy alone statistically reduced levels in both groups of total oxidant status (p < 0.001 in both groups) and lipid peroxidation (p = 0.016 in RIPE group and p < 0.001 in RIPENAC group) (**Figures 3A, B**), whereas levels of DNA oxidation were just affected by TB antibiotic regimen in the RIPENAC group (p = 0.025) (**Figure 3C**). Interestingly, patients receiving NAC displayed a more remarkable reduction in lipid peroxidation (p < 0.001) and DNA oxidation (p = 0.048) values compared to those from the control group, whereas total oxidant status were not significantly affected by NAC-adjunct therapy (**Figures 3A–C**). Importantly, levels of important antioxidant molecules such as catalase and ferritin-H were altered by TB drug therapy only on the group with NAC-adjunct treatment (p = 0.02 and p = 0.03, respectively). Superoxide dismutase activity was not altered in any time or group (**Figures 4A–C**). In addition, we failed to find difference in total levels of catalase, ferritin-H and superoxide dismutase as a consequence of NAC treatment when both groups of patients were compared at the same time-point (60 days) (**Figures 4A–C**).

Additionally, we evaluated patients who received NAC therapy, stratifying them in two groups: (i) patients who persisted with positive culture at month 2 or died (n = 8) and (ii) those who did convert culture to negative and survived (n = 10). No significant differences were found between these two subgroups, suggesting that this therapy regimen is not associated with death or persistence of Mtb detection in cultures after 60 days of treatment (**Table S3**). Further analyses comparing RIPENAC and RIPE groups with regard to clinical outcomes were carried out in our previous study, and again no associations between use of NAC and unfavorable/adverse outcomes were observed (16). Altogether these findings suggest that co-administration of NAC with anti-TB treatment causes changes on the inflammatory profile of the patients, with decreased levels of systemic oxidation by rising levels of GSH.

## Discussion

A number of studies have pointed out that optimal host immune response against pathogens can be affected by oxidative stress (38–41). In general, redox homeostasis perturbance is marked by an imbalance between free radicals and antioxidant molecules which can lead to cell death and tissue damage. Lungs are continuously exposed to many exogenous oxidative compounds and/or pathogens requiring a very efficient antioxidant mechanism to avoid tissue damage and exacerbation of the host immune response (42). NAC is widely used as a potent oxidative stress modulator since this compound is a precursor for glutathione, an important antioxidant molecule in promoting redox homeostasis (10). NAC cytoprotective property is clinically evident during TB and HIV chemotherapy preventing drug-induced hepatotoxicity (43–45). In this study, we aimed to assess the impact of NAC-adjunctive treatment on host immune response and redox homeostasis in hospitalized HIV/TB patients. Considering that these patients exhibit worse health status compared to non-hospitalized subjects, it is paramount to devote maximum attention to pursuing new adjunctive therapies and thus improving the current therapy protocol employed to this particular group of individuals. Since these patients are admitted to a hospital environment, it is straightforward to continuously evaluate changes in their health status which also include parameters associated with immune response and redox homeostasis during the adjunct treatment.

Recently, we published a report describing the safety of NAC treatment in hospitalized HIV/TB patients which were daily monitored (16). By the end of that study, we were able to conclude that NAC administration was not unsafe for the enrolled patients and also displayed a strong capacity in reducing Mtb sputum culture positivity (16). These exciting findings encouraged us to investigate the effect of NAC treatment in modulating oxidative stress and host immune response against Mtb. We randomly arranged these hospitalized HIV/TB patients in two independent groups, here referred to as RIPE (under TB antibiotic therapy) and RIPENAC (receiving NAC in addition to TB antibiotic treatment). Samples were collected from these patients at the baseline and 60 days after NAC-adjunctive therapy to follow the changes in their immune response and oxidative stress. The large number of inflammatory mediators analyzed in this study showed a substantial change in IL-1RA, IL-17A, and VEGF levels upon NAC administration compared to that in the control group, suggesting a change in the pattern of pro-inflammatory response. This finding likely means that NAC therapy may amplify the host benefits observed during anti-TB therapy. In fact, it is known that enhanced production of type I IFNs are responsible for the susceptibility to Mtb infection, mainly IL-1RA that is an important mediator of driven susceptibility *in vivo* (39). In contrast, higher levels of IL-17A are associated with a development of an early protective immunity and the formation of mature granulomas in Mtb infection (46–48). Thus, the decrease in levels of IL-1RA and increase of IL-17A in patients who received NAC therapy, shows a protective factor of susceptibility to infection. Although a decrease in plasma VEGF levels is commonly observed during antitubercular treatment (49, 50), it is known that decreased oxidative stress and inflammation stimulate VEGF expression (51). Thus, the increase observed in our study over the treatment time may be associated with a marked decrease in oxidation levels. Future studies will be carried out to assess whether this is a lasting or temporary effect. Furthermore, an unsupervised hierarchical cluster analysis revealed four distinct clusters of inflammatory markers highlighting changes in the host immune response in patients receiving NAC. Interestingly, Islamoug et al. (2018) examined the capacity of liposomal glutathione in modulating granulomatous response against TB infection and clearly showed that glutathione supplementation into human PBMC cultures diminishes the production of pro-inflammatory cytokines including IL-6 and TNF-α, as well as prevented mycobacterial proliferation (52). Thus, the modulation of host immune response to optimal levels is crucial for the control of Mtb infection avoiding induction of immunopathology.

Another factor associated with inflammatory responses is an imbalance of oxidant and antioxidant levels. Several studies have suggested that exacerbated accumulation of pro-oxidant molecules can be detrimental for the host, leading to unfettered production of pro-inflammatory cytokines, a phenomenon known as “cytokine storm”. This phenomenon has been described in several respiratory infectious diseases which usually progress to acute respiratory distress syndrome. This cytokine storm phenomenon has been extensively revised during the most recent world pandemic caused by SARS-Cov-2 infection, the pathogenic agent of COVID-19 (53, 54). This disease revealed an urgent need for new therapeutic strategies to control the exacerbation of host immune response preventing tissue damage and consequently organ failure. In TB, the elevated ROS production is associated with disease severity and also has been linked with lowered levels of GSH (55). Of note, the depletion of GSH is also found in PLWHIV (56). Interestingly, PBMCs isolated from PLWHIV has been reported to display elevated host immune resistance to Mtb infection when GSH precursor was administrated to the cell cultures (56). Moreover, in animal models of pulmonary TB, NAC treatment is related to significant effects in restoring antioxidant capacity, minimizing cellular necrosis and mycobacterial proliferation (11). These findings suggest that adjunct NAC treatment may aid host response against pathogens through two mechanisms, by preventing cytokine storm and by improving host cell ability to kill the pathogen.

Since GSH has been suggested as an efficient strategy in regulating oxidative stress and tissue damage (13, 14), we wanted to investigate, in this study, whether NAC administration to hospitalized HIV/TB patients could serve as a reliable adjunctive intervention targeting improvement in GSH levels, reducing pro-oxidant molecules and preventing cell damage. After 60 days of NAC administration, we found that RIPENAC patients displayed elevated plasma levels of GSH along with increased levels of total antioxidant status and reduced levels of both lipid peroxidation and DNA oxidation compared to RIPE group at the same time-point. Interestingly, we failed to find a significant difference in other antioxidant molecules such as superoxide dismutase activity, catalase and ferritin-H when the RIPENAC group was compared to RIPE patients. However, NAC treatment enhanced levels of catalase and ferritin-H only in RIPENAC patients at 60 days compared to the baseline levels.

This present investigation has some limitations. The number of study participants enrolled to the RIPENACTB trial was relatively small, which prevented us to perform additional analyses in subgroups of patients stratified based on mycobacterial loads in sputum samples. Moreover, we recruited hospitalized with HIV-associated TB and thus the findings reported here may not be extrapolated to non-severe patients in outpatient clinics. Due to the fact that the study population was composed by patients with drug-sensitive TB, the effect of NAC treatment may also not be applicable to cases of multidrug-resistant Mtb, which have special need to host adjunctive therapies. The effects of NAC therapy were not investigated for long-term outcomes, and it is possible that reduction of oxidative stress could lead to improve lung function post treatment. Regardless of such limitations, our findings add to the current knowledge in the field by demonstrating that NAC may be a relevant candidate for adjunct therapy in TB. Though detailed characterization of the effects of NAC on systemic inflammation and oxidative stress in peripheral blood, our study presents the basis to support implementation of NAC therapy in large multicentric clinical trials. The results presented here clearly show that the group of patients undertaking NAC exhibited significant increase in GSH levels and in total antioxidant status while displaying substantial reduction in lipid peroxidation compared to the control group. If the results presented here are validated in such settings, systematic use of NAC adjunct therapy could be considered to alleviate the TB burden and improve clinical management of PLWHIV.

## Data Availability Statement

The raw data supporting the conclusions of this article will be made available by the authors, without undue reservation.

## Ethics Statement

The studies involving human participants were reviewed and approved by Ethics Review Committee of Fundação de Medicina Tropical Dr Heitor Vieira Dourado (FMT-HVD) (protocol study number: 60219916.5.0000.0005). The patients/participants provided their written informed consent to participate in this study.

## Author Contributions

IS, ML, FB-M, WM, EA, VS, BA, and MC-S contributed to conception and design of the study. ML, VP, FB-M, WM, VS, BA, and MC-S performed the data curation. ML, BB-D, AA, WM, VS, MA-P, BA, and MC-S processed and analyzed the data. MA-P and BA worked on data visualization. EA, MA-P, and BA wrote the first draft of the manuscript. All authors contributed to the article and approved the submitted version.

## Funding

This work was supported by *Cooperação Interfederativa do Amazonas* (Interfam), funded by the Brazilian Ministry of Health. The study was partially supported by the Intramural Research Program of the Fundação Oswaldo Cruz, Brazil. The work of BA was supported by the National Institutes of Health [grant numbers: U01AI115940, U01AI069923]. ML, WM, and BA are senior fellows from the Conselho Nacional de Desenvolvimento Científico e Tecnológico (CNPq), Brazil. EA is a Research Fellow from the National Institute of Allergy and Infectious Diseases, National Institutes of Health. MA-P received a fellowship from Coordenação de Aperfeiçoamento de Pessoal de Nível Superior, Brazil [Finance code: 001].

## Conflict of Interest

The authors declare that the research was conducted in the absence of any commercial or financial relationships that could be construed as a potential conflict of interest.
