# Supplementary material for: Adjunct N-Acetylcysteine Treatment in Hospitalized Patients With HIV-Associated Tuberculosis Dampens the Oxidative Stress in Peripheral Blood: Results From the RIPENACTB Study Trial
Source: Front Immunol. 2021 Feb 4;11:602589. doi: 10.3389/fimmu.2020.602589 (PMC7889506; doi:10.3389/fimmu.2020.602589)
Supplement: Supplementary file 1 [file Table_1.docx]

Adjunct N-acetylcysteine treatment in hospitalized patients with HIV-associated tuberculosis dampens the oxidative stress in peripheral blood: Results from the RIPENACTB Study trial

Izabella P. Safe, Eduardo P. Amaral, Mariana Araújo-Pereira, Marcus V. G. Lacerda, Vitoria S. Printes, Alexandra B. Souza, Francisco Beraldi-Magalhães, Wuelton M. Monteiro, Vanderson S. Sampaio, Beatriz Barreto-Duarte, Alice M. S. Andrade, Renata Spener-Gomes, Allyson Guimarães Costa, Marcelo Cordeiro-Santos, Bruno B. Andrade

Supplementary Material


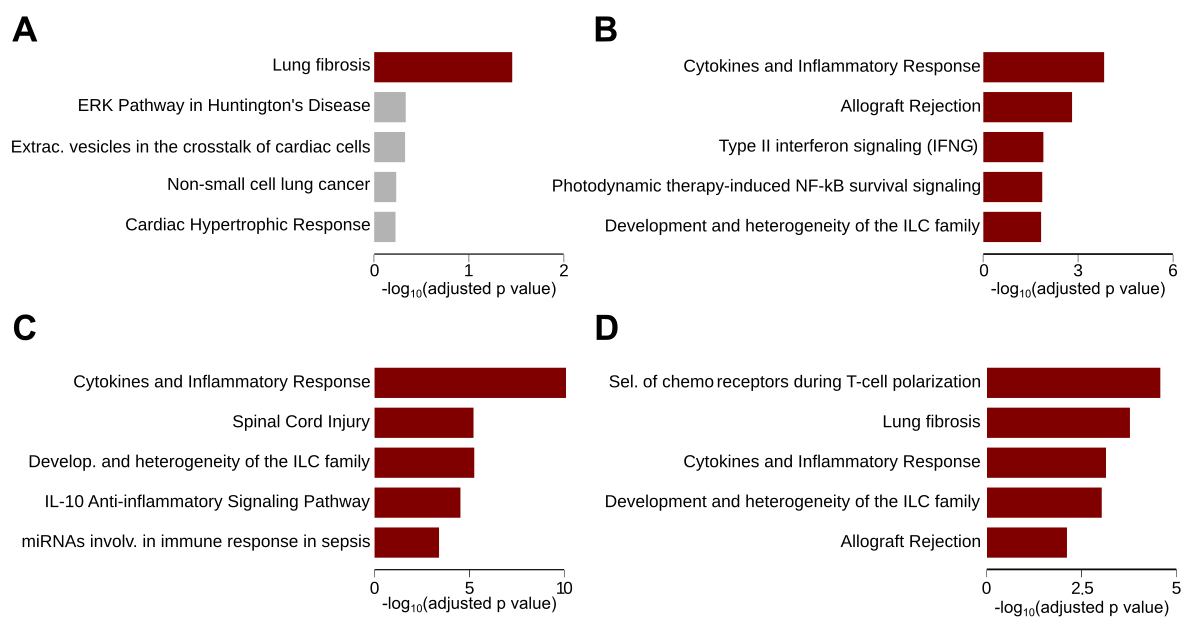
**Figure S1. Enrichment analysis of unsupervised biomarker clusters.** The enrichment was performed using EnrichR web tool, against WikiPathways base. The first five pathways with the lowest adjusted p values for the clusters c1 (A), c2 (B), c3 (C) and c4 (D) are represented in the graphs. Red bars indicated pathways with adjusted p < 0.05.

**Table S1. Concentrations of biomarkers at baseline (before treatment).**

| Parameter | RIPE (n=21) | RIPENAC (n=18) | P-value |
| --- | --- | --- | --- |
| EGF | 39 (26.5-188.5) | 39 (17-136.25) | 0.474 |
| EOTAXIN/CCL11 | 271 (141-343.75) | 272 (176.25-326.75) | 0.976 |
| GCSF | 65.5 (39.75-81) | 94 (60-150.25) | 0.050 |
| GMCSF | 33 (22.5-36.75) | 30.5 (24-33) | 0.615 |
| IFN-α2 | 27 (21.75-35.5) | 24 (17.5-27) | 0.108 |
| IFN-γ | 38 (30.75-53.5) | 66 (31.25-89.5) | 0.286 |
| IL-10 | 49 (35-64) | 62.5 (45-132.25) | 0.121 |
| IL-12p40 | 17 (14.5-25.5) | 22 (16-24.5) | 0.976 |
| IL-12p70 | 28 (22.5-34.25) | 30.5 (25-32.5) | 0.529 |
| IL-13 | 21 (17-26.75) | 19 (17.5-24.25) | 0.725 |
| IL-15 | 53 (38.75-68) | 61 (44-85.25) | 0.231 |
| IL-17A | 20.5 (18.75-26.75) | 23 (20-26.5) | 0.86 |
| IL-1RA | 36 (28.25-58) | 45 (40.5-127.5) | 0.050 |
| IL-1α | 91.5 (62.75-144.75) | 138 (112-189.25) | **0.034** |
| IL-1β | 31 (25-35.75) | 25.5 (21.75-36.5) | 0.629 |
| IL-2 | 29 (24.5-37.5) | 27 (23.75-29) | 0.128 |
| IL-3 | 26 (20-32.75) | 26 (19.25-29.5) | 0.529 |
| IL-4 | 15 (13-17) | 15 (13.25-16.5) | 0.953 |
| IL-5 | 19.5 (16-24) | 15 (13.25-25.5) | 0.364 |
| IL-6 | 172 (85.75-424) | 404 (90.5-1185.5) | 0.225 |
| IL-7 | 32 (27.5-40.75) | 35 (31.5-37) | 0.883 |
| IL-8 | 326 (159.75-563.75) | 421.5 (193.25-921.5) | 0.369 |
| IP10/CXCL10 | 6853 (4790-9238) | 9772 (7965-12775) | **0.025** |
| MCP1/CCL2 | 1298 (920.5-2369.5) | 1658 (548-3428.5) | 0.772 |
| MIP1A/CCL3 | 41 (29.5-56.25) | 62 (29-97) | 0.335 |
| MIP1B/CCL4 | 33 (29.25-44.5) | 32.5 (26.5-42.5) | 0.837 |
| TNF-α | 143 (122-252) | 252.5 (134.75-454) | 0.184 |
| TNF-β | 23 (18-26) | 23 (18.5-25.75) | 0.86 |
| VEGF | 25.5 (22.5-33.5) | 27 (21-30) | 0.73 |
| LTB4 | 31689(30582-42129) | 34407(25293-38160) | 0.73 |
| PGE2 | 201(105-520.9) | 146(80.4-205) | 0.08 |

**Table note:**

Data are shown as median and interquartile (IQR) range of plasma concentration (pg/mL).

P-values were calculated using Wilcoxon rank test (continuous variables). Bold-type font indicates statistical significance. The cytokines were measured as described in Methods.

**Table S2. Concentrations of biomarkers at baseline and after 60 days of treatment.**

|  | RIPE (n=21) | |  | RIPENAC (n=18) | |  | P-value |
| --- | --- | --- | --- | --- | --- | --- | --- |
|  | Day 0 | Day 60 | P-value Δ(d60-d0) | Day 0 | Day 60 | P-value Δ(d60-d0) |  |
| **EGF** | 39 (26.5-188.5) | 121 (21.6-441.6) | 0.320 | 39 (17-136.25) | 89.5 (53-225.5) | 0.194 | 0.863 |
| **EOTAXIN/CCL11** | 271 (141-343.75) | 392 (222.8-546.5) | 0.110 | 272 (176.25-326.75) | 254 (162.8-402.4) | 0.675 | 0.311 |
| **GCSF** | 65.5 (39.75-81) | 42.75 (32.3-53.8) | 0.144 | 94 (60-150.25) | 55.5 (38-64.4) | 0.037 | 0.506 |
| **GMCSF** | 33 (22.5-36.75) | 25 (25-30.4) | 0.339 | 30.5 (24-33) | 25 (23.6-27.8) | 0.127 | 0.781 |
| **IFN-α2** | 27 (21.75-35.5) | 24 (18.4-27.1) | 0.208 | 24 (17.5-27) | 23.5 (21-28.9) | 0.477 | 0.153 |
| **IFN-γ** | 38 (30.75-53.5) | 25.5 (19.5-36.6) | **0.033** | 66 (31.25-89.5) | 33.75 (25.5-52.3) | 0.096 | 0.521 |
| **IL-10** | 49 (35-64) | 40 (31.9-70.4) | 0.641 | 62.5 (45-132.25) | 47 (38.5-62.5) | 0.224 | 0.212 |
| **IL-12p40** | 17 (14.5-25.5) | 17 (14.5-19) | 0.394 | 22 (16-24.5) | 19.5 (16.8-23.8) | 0.123 | 0.430 |
| **IL-12p70** | 28 (22.5-34.25) | 33.25 (24.5-43.4) | 0.350 | 30.5 (25-32.5) | 32 (29-37.5) | 0.761 | 0.144 |
| **IL-13** | 21 (17-26.75) | 18.75 (16-21.9) | 0.273 | 19 (17.5-24.25) | 20 (18.8-23) | 0.462 | 0.373 |
| **IL-15** | 53 (38.75-68) | 44.5 (38.3-51) | 0.209 | 61 (44-85.25) | 46 (42-54.1) | 0.065 | 0.931 |
| **IL-17A** | 20.5 (18.75-26.75) | 19.5 (18.4-28.1) | 0.760 | 23 (20-26.5) | 27.5 (23.5-30.3) | **0.050** | **0.012** |
| **IL-1RA** | 36 (28.25-58) | 31 (26.3-56.3) | 0.655 | 45 (40.5-127.5) | 37.25 (32.6-39.1) | **0.020** | **0.027** |
| **IL-1α** | 91.5 (62.75-144.75) | 79 (61.3-165.1) | 0.935 | 138 (112-189.25) | 103.5 (82.8-142.1) | 0.110 | 0.470 |
| **IL-1β** | 31 (25-35.75) | 21.75 (20.4-26.3) | 0.088 | 25.5 (21.75-36.5) | 25.75 (20.9-29.6) | 0.776 | 0.759 |
| **IL-2** | 29 (24.5-37.5) | 26.5 (22.5-31.1) | 0.361 | 27 (23.75-29) | 28 (25.9-29) | 0.515 | 0.070 |
| **IL-3** | 26 (20-32.75) | 24 (18.8-28.6) | 0.351 | 26 (19.25-29.5) | 27.5 (24-30) | 0.309 | 0.274 |
| **IL-4** | 15 (13-17) | 14.5 (12-16.3) | 0.514 | 15 (13.25-16.5) | 15.5 (12.9-18.3) | 0.699 | 0.073 |
| **IL-5** | 19.5 (16-24) | 18 (15.8-25.4) | 0.871 | 15 (13.25-25.5) | 23.25 (19-26.8) | 0.095 | 0.062 |
| **IL-6** | 172 (85.75-424) | 46.75 (34-92.5) | **0.007** | 404 (90.5-1185.5) | 93.25 (71.3-117.5) | 0.093 | 0.931 |
| **IL-7** | 32 (27.5-40.75) | 27.5 (24.8-32) | 0.056 | 35 (31.5-37) | 32 (26.4-36.5) | 0.515 | 0.099 |
| **IL-8** | 326 (159.75-563.75) | 264.5 (121.8-553.8) | 0.675 | 421.5 (193.25-921.5) | 446.75 (266-612) | 0.984 | 0.470 |
| **IP10/CXCL10** | 6853 (4790-9238) | 6567 (4972-10687) | 0.984 | 9772 (7965-12775) | 7683 (555-9695) | **0.048** | 0.863 |
| **MCP1/CCL2** | 1298 (920.5-2369.5) | 1198.5 (539-2319.1) | 0.367 | 1658 (548-3428.5) | 844.5 (326-1199) | 0.120 | 0.070 |
| **MIP1A/CCL3** | 41 (29.5-56.25) | 34 (29-69.8) | 0.855 | 62 (29-97) | 68.5 (43.8-171) | 0.490 | 0.885 |
| **MIP1B/CCL4** | 33 (29.25-44.5) | 31.5 (26.5-37.8) | 0.516 | 32.5 (26.5-42.5) | 47.5 (35.5-52.1) | 0.118 | 0.418 |
| **TNF-α** | 143 (122-252) | 186.5 (129.8-295.4) | 0.887 | 252.5 (134.75-454) | 250.5 (184-322.8) | 0.921 | 0.931 |
| **TNF-β** | 23 (18-26) | 21.5 (16.3-23) | 0.247 | 23 (18.5-25.75) | 23.5 (21.8-28.9) | 0.339 | 0.189 |
| **VEGF** | 25.5 (22.5-33.5) | 24.75 (20.8-30.8) | 0.383 | 27 (21-30) | 30.25 (25.8-34.9) | 0.187 | **0.043** |
| **LTB4** | 31689(30582-42129) | 33713(22293-36185) | 0.320 | 34407(25293-38160) | 36840 (24699-41039) | 0.194 | 0.402 |
| **PGE2** | 201(105-520.9) | 435 (232-552) | 0.110 | 146(80.4-205) | 362 (145.6-415.7) | 0.675 | 0.172 |

**Table note:**

Data are shown as median and interquartile (IQR) range. Values represent concentration (pg/mL). P-values were calculated using Wilcoxon rank test (continuous variables). The third p-value was performed to compare delta variation (D60-D0) of each biomarker between the clinical groups.

Bold-type font indicates statistical significance.

The cytokines were measured as described in Methods.

**Table S3. Concentrations of biomarkers at baseline in NAC patients by culture conversion or death.**

|  | Culture conversion or survivor (n=10) | Without culture conversion/Death (n=8) | P-value |
| --- | --- | --- | --- |
| **EGF** | 39.0 (19.0 - 136) | 66.5 (16.5 - 147) | 1.000 |
| **EOTAXIN/CCL11** | 272 (191 - 343) | 262 (131 - 308) | 0.409 |
| **GCSF** | 82.5 (60.0 - 116) | 119 (74.4 - 195) | 0.457 |
| **GMCSF** | 30.0 (23.5 - 32.5) | 31.2 (27.8 - 36.6) | 0.432 |
| **IFN-α2** | 24.0 (20.5 - 27.5) | 18.8 (15.8 - 25.8) | 0.231 |
| **IFN-γ** | 46.0 (28.2 - 85.0) | 79.8 (48.2 - 123) | 0.283 |
| **IL-10** | 46.0 (44.0 - 71.8) | 129 (93.5 - 170) | 0.083 |
| **IL-12p40** | 30.5 (25.2 - 32.0) | 29.5 (24.0 - 33.8) | 0.868 |
| **IL-12p70** | 23.5 (15.5 - 25.0) | 21.0 (16.5 - 22.2) | 0.408 |
| **IL-13** | 20.0 (18.5 - 22.2) | 18.5 (16.5 - 35.8) | 0.901 |
| **IL-15** | 56.0 (45.5 - 82.0) | 61.0 (45.0 - 89.6) | 0.836 |
| **IL-17A** | 22.0 (20.0 - 27.2) | 23.0 (19.0 - 26.1) | 0.836 |
| **IL-1RA** | 43.5 (36.8 - 114) | 91.8 (43.8 - 155) | 0.457 |
| **IL-1α** | 133 (109 - 140) | 178 (145 - 204) | 0.16 |
| **IL-1β** | 25.0 (19.2 - 33.0) | 30.0 (24.4 - 37.0) | 0.508 |
| **IL-2** | 27.0 (23.8 - 29.0) | 26.5 (23.5 - 29.5) | 0.901 |
| **IL-3** | 28.0 (20.0 - 30.0) | 23.5 (18.8 - 29.0) | 0.32 |
| **IL-4** | 15.0 (13.8 - 16.0) | 15.0 (11.4 - 17.6) | 0.934 |
| **IL-5** | 17.0 (14.2 - 25.5) | 14.2 (9.88 - 25.5) | 0.432 |
| **IL-6** | 183 (75.5 - 1647) | 902 (346 - 1083) | 0.509 |
| **IL-7** | 35.0 (31.5 - 39.2) | 34.8 (30.6 - 37.0) | 0.836 |
| **IL-8** | 312 (193 - 922) | 426 (228 - 967) | 1.000 |
| **IP10/CXCL10** | 9250 (7104 - 10586) | 12330 (10896 - 14574) | 0.069 |
| **MCP1/CCL2** | 1658 (631 - 3320) | 1393 (493 - 3481) | 0.804 |
| **MIP1A/CCL3** | 47.5 (29.0 - 78.5) | 79.8 (37.5 - 145) | 0.535 |
| **MIP1B/CCL4** | 32.0 (26.0 - 41.2) | 33.2 (29.6 - 52.1) | 0.563 |
| **TNF-α** | 218 (124 - 372) | 443 (241 - 497) | 0.16 |
| **TNF-β** | 23.0 (18.5 - 25.5) | 22.0 (18.5 - 26.4) | 0.868 |
| **VEGF** | 27.0 (25.5 - 29.2) | 25.5 (17.9 - 32.1) | 0.803 |
| **LTB4** | 35859 (31451 - 39167) | 30073 (22074 - 36421) | 0.342 |
| **PGE2** | 184 (126 - 236) | 97.1 (0.07 - 147) | 0.112 |

**Table note:**

Data are shown as median and interquartile (IQR) range. Values represent concentration (pg/mL). Concentrations for each biomarker were compared between the clinical groups using the Mann-Whitney *U* test (continuous variables).

The cytokines were measured as described in Methods.
